# Transforming Veteran Rehabilitation Care: Learnings from a Remote Digital Approach for Musculoskeletal Pain

**DOI:** 10.3390/healthcare12151518

**Published:** 2024-07-31

**Authors:** Anabela C. Areias, Dan Doverspike, Daniel F. Brostek, Dora Janela, Michael S. Erwin, John M. Pinter, James R. Ficke, Fabíola Costa

**Affiliations:** 1 Clinical Research, Sword Health, Inc., Draper, UT 84020, USA; a.areias@swordhealth.com (A.C.A.); d.janela@swordhealth.com (D.J.); 2 Government Programs, Sword Health, Inc., Draper, UT 84020, USA; d.doverspike@swordhealth.com; 3 Team Red, White, and Blue, Inc., Floyds Knobs, IN 47119, USA

**Keywords:** musculoskeletal pain, physical therapy, pain management, telerehabilitation, eHealth, veterans

## Abstract

While musculoskeletal pain (MSP) stands as the most prevalent health condition among Veterans, timely and high-quality care is often hindered due to access barriers. Team Red, White & Blue (Team RWB), a nonprofit organization dedicated to promoting a healthier lifestyle among Veterans, aimed to assess innovative approaches to veteran care. This is a single-arm pilot study investigating the feasibility, clinical outcomes, engagement, and satisfaction of a remote multimodal digital care program among Veterans with MSP. The impact of deployment experience on outcomes was explored as a secondary aim. From 75 eligible Veterans, 61 started the program, reporting baseline pain frequently comorbid with mental distress. Program acceptance was suggested by the high completion rate (82%) and engagement levels, alongside high satisfaction (9.5/10, SD 1.0). Significant improvements were reported in all clinical outcomes: pain (1.98 points, 95%CI 0.13; 3.84, *p* = 0.036); mental distress, with those reporting at least moderate baseline depression ending the program with mild symptoms (8.50 points, 95%CI: 6.49; 10.51, *p* = 0.012); daily activity impairment (13.33 points, 95%CI 1.31; 25.34, *p* = 0.030). Deployed Veterans recovered similarly to their counterparts. Overall, the above results underscore the potential of a remote digital intervention to expand Veterans’ access to timely MSP care.

## 1. Introduction

In the realm of military service, Veterans often contend with a myriad of health adversities impacting their quality of life. Musculoskeletal pain (MSP) stands out as the most prevalent and costly of all disorders managed in Veterans Health Administration (VHA) facilities, being therefore recognized as a top priority [1,2]. From the 20 million Veterans residing nationwide [3], about 66% suffer from MSP [4], with this population experiencing three-fold higher odds of developing severe pain compared with non-veterans [4]. As an example, back and neck pain have been reported as more predominant conditions in veterans than non-veterans (21.6% vs. 16.7% and 27.7% vs. 21.4%, respectively) [4].

Chronic MSP and mental distress comorbidity has been extensively documented [5], with a notably higher prevalence observed among the veteran population [6,7]. Traumatic experiences during military service may contribute to this increased likelihood [8].

A range of treatment options span from conservative non-pharmacologic care to opioids, injections, or even surgical procedures. Notably, specialized care encompassing multimodal interventions composed of exercise, education, and behavioral change is universally recommended as the first-line approach [9,10,11,12], including by VHA [13]. Although 66% of Veterans experience MSP [4], only 37% undergo physical or occupational therapy care [14], despite clinical guidelines supporting physical therapy as a first-line treatment for chronic pain [15,16]. One-quarter of Veterans live in rural areas, compounding the access challenges even further [17]. Additionally, disbelief about non-pharmacological treatment, poor social support, and isolation have hindered many from timely and proper care [18]. Opioid dependence is very common among Veterans with chronic pain [19], with the VHA expanding on initiatives prompting non-pharmacological treatment to reduce opioid prescriptions [9]. Thus, it has become increasingly important to identify new innovative approaches to veteran care.

Remote care has emerged as a potential solution to improve accessibility to proper care, and it has been increasingly considered for veteran populations [20,21,22]. Early studies evaluated the potential of hybrid MSP care (combining remote with in-person consultations) [20], or remote interdisciplinary care focused on psychological care, which included exercise [21], in this particular population. Levy et al. [20] highlighted that alongside positive clinical outcomes, Veterans undergoing hybrid MSP care saved 2774.7 travel miles, 46.3 h of driving time, and USD 1151.50 in travel reimbursement, as a testament to the potential cost-effectiveness of remote care. However, the application of telerehabilitation for MSP management remains greatly underexplored among the veteran community, especially regarding fully remote interventions. Currently, it is unknown whether this care modality can effectively promote clinical outcomes and patient engagement.

Previously, we reported about the effectiveness of multimodal digital care programs (DCPs) integrating exercise, education, and cognitive behavioral therapy (CBT), where each patient is assigned to a doctor of physical therapy responsible for their close monitoring. This DCP was validated in several musculoskeletal conditions [23,24], including in populations with different ages [25], race/ethnicity backgrounds [26], and socioeconomic status [27], reflecting that a therapeutic alliance is possible among completely remote programs to promote significant clinical improvement regardless of the background. This study arose from a collaboration with Team Red, White & Blue (Team RWB), a nonprofit organization dedicated to promoting a healthier lifestyle among over 268,000 of America’s Veterans and supporters through a variety of programs and initiatives. To assess innovative approaches to veteran care, this single-arm pilot study aims to assess the feasibility, clinical outcomes, and engagement of a remote multimodal DCP with biofeedback in a veteran population (enrolled through the Team RWB initiative) who suffered from MSP in either spine or upper and lower limbs. We hypothesized that Veterans following this intervention would report significant improvements in all clinical outcomes, including pain, mental health, productivity, and activities of daily living impairment. As a secondary aim, we evaluated if deployed Veterans would have similar recovery trajectories to non-deployed Veterans. By exploring the feasibility, acceptance, and outcomes of this DCP, we will gain insights that enable comparisons to other DCP modalities (e.g., not fully remote), and gather knowledge to establish and support future research steps, as well as inform clinical practice and healthcare policies about alternative and timely delivery modes for this particular population.

## 2. Materials and Methods

### 2.1. Study Design

The study aims to investigate the feasibility of a multimodal digital care program (DCP) in a Veteran cohort with musculoskeletal pain (MSP). This pilot study constitutes an ad hoc analysis of an ongoing decentralized single-arm clinical trial investigating clinical and engagement-related outcomes after a DCP in patients with MSP. The trial was prospectively approved by the Advarra Institutional Review Board (Pro00063337) and registered in ClinicalTrials.gov (NCT05417685) on 14 June 2022. The home-based DCP was delivered between 12 December 2023 and 2 May 2024.

### 2.2. Population

This pilot trial was conducted in collaboration with Team RWB, under their initiatives to advance veteran health and wellness by supporting innovative research as well. Recruitment was facilitated by Team RWB, focusing on Veterans based in the United States (US) who were experiencing either acute or chronic musculoskeletal pain (MSP) in the spine and upper or lower limbs. Only participants with cognitive stability (i.e., able to autonomously follow directions and simple motor commands) were eligible to participate in the program. These Veterans were invited to apply for Sword Health’s DCP, located in Draper, UT, USA, through a specialized website.

Exclusion criteria included (1) having a health condition (e.g., cardiac, respiratory) that prevented at least 20 min of light to moderate exercise; (2) receiving treatment for active cancer; (3) experiencing rapidly progressive loss of strength and/or numbness in the arms or legs, or an unexplained change in bowel or urinary function in the previous two weeks; and (4) failure to engage in at least two exercise sessions. Informed consent was obtained from all participants.

### 2.3. Intervention

The DCP consisted of a digitally delivered intervention that included exercise, education, and cognitive behavioral therapy (CBT) for up to 12 weeks according to clinical practice guidelines [10,11,12] (further details in Supplementary Table S1), as described elsewhere [23,24,25,26,27]. After enrolling through a dedicated website, patients filled out a baseline form with demographic and clinical characteristics, and chose their physical therapist (PT) who was responsible for program tailoring and monitoring. Further medical history was collected subsequently during the onboarding, where goals were established through shared decision-making. In case of flagged severe comorbid mental conditions, patients were informed of available care resources. Personalized exercise sessions were performed independently at the patients’ convenience (3 sessions/week were recommended) using an FDA-listed class II medical device that comprised a mobile app on a dedicated tablet, a motion tracking system, and a cloud-based portal. Exercises were displayed on the tablet, whose motion tracking allowed for real-time video and audio biofeedback on performance. A cloud-based portal allowed the assigned PT to remotely monitor and adjust treatment asynchronously. A tailored educational component and CBT were also provided as needed [11,12,28,29]. Educational components followed current clinical guidelines and research, including topics focused on anatomy, physiology, symptoms, evidence-based treatments, fear avoidance, and active coping skills. The CBT program was based on mindfulness, acceptance and commitment therapy, empathy-focused therapy, fear-avoidance behavior, and constructive coping. This was administered on an as-needed basis as self-guided interactive modules delivered through the smartphone app. PHQ-9 and GAD-7 scores were used not only to guide the intervention approach but also to direct members to psychological and/or psychiatric care when needed, following the US Department of Health and Human Services guidelines. Bi-directional communication with the assigned PT was ensured through a built-in secure chat within a smartphone app. Participants who did not engage in any exercise session for 30 consecutive days were considered dropouts.

### 2.4. Demographic Data

Demographic data collected at onboarding included age, race/ethnicity, body mass index (BMI), gender, symptomatic body region, educational level, employment status and geographic location (rural vs. urban). Additional demographic information was made available by Team RWB, namely branch of service, deployment experience (yes/no), and Former Military Officer (yes/no). Geographic locations were established by coding each veteran to a specific rural–urban commuting area (RUCA) according to their ZIP codes [30] (urban = 1 to 3 and rural = 4 to 10).

### 2.5. Clinical Outcomes

Outcomes were collected at baseline and at the following sessions until discharge: 9, 14, 19, and 24 sessions. Mean changes were calculated between baseline and treatment end. All Patient Reported Outcome Measures (PROMs) used were previously validated elsewhere [31,32,33,34,35].

Primary outcome was self-reported pain, using the Numerical Pain Rating Scale (NPRS), through the following question: “Please rate your average pain over the last 7 days” from 0 (no pain at all) to 10 (worst pain imaginable)” [31,36]. Minimum clinically important change (MCIC) of at least 2 points between baseline and treatment end was considered as a clinically relevant improvement, following IMMPACT guidelines [37].

Secondary outcomes included the following clinical and engagement outcomes:

Mental health: Generalized Anxiety Disorder 7-item (GAD-7) scale (range 0–21) to assess anxiety [32,34], and Patient Health 9-item questionnaire (PHQ-9) (range 0–27) to assess depression [33,34]. Higher scores indicate worse symptoms.

Work Productivity and Activity Impairment (WPAI) for general health questionnaire evaluated on employed participants to assess overall work impairment (WPAI overall: total presenteeism and absenteeism from work), presenteeism (WPAI work), absenteeism (WPAI time), and activity impairment (WPAI activity) [35]. Higher scores indicate greater impairment (0–100).

Engagement: measured through the following: (A) completion of the program (considered as the retention rate); (B) total number of completed exercise sessions over the program; (C) overall satisfaction through the following question: “On a scale from 0 to 10, how likely is it that you would recommend this intervention to a friend or neighbor?”.

### 2.6. Safety and Adverse Events

Patients were instructed to report any adverse events when they occurred to their PT. Additionally, pain and fatigue scores (graduated from 0 to 10) were collected at the end of each session and monitored remotely by the PT.

### 2.7. Data Availability

All relevant data are included in the article or available in Supplementary Material Data. De-identified data and analysis codes may be provided upon reasonable request to the corresponding author.

### 2.8. Sample Size

Previous studies on MSP interdisciplinary rehabilitation in a veteran population have reported effect sizes for pain ranging from 0.94 [38] to 0.35 [39] from baseline to treatment end. To provide a balanced approach considering this range, a medium effect size of 0.5 was selected [40]. Using G*Power (version 3.1.9.7.), with an 80% power and a two-sided 0.05 significance level, the required sample size was calculated to be 34 participants. To mitigate a potential attrition of 20%, as previously described in telerehabilitation, a total of at least 41 participants were required for this pilot study.

### 2.9. Statistical Analysis

Demographics, clinical data, and engagement metrics were reported through descriptive statistics, with continuous variables described as mean (standard deviation) and categorical variables as frequencies (percentage). Baseline differences between completers and non-completers (i.e., those who dropped out) and between deployed and non-deployed Veterans were assessed through chi-squared tests or Fisher’s exact test (depending on sample size) for categorical variables, and independent sample t-tests for continuous variables.

Latent growth curve analysis (LGCA) assessed outcome changes across the program, following both an intention-to-treat and a per-protocol analysis. LGCA provides an estimate of the average trajectory and individual variation based on each individual piece of data and sessions performed. This methodology is estimated as a structural equation model [41], with the advantages of providing a measure of fitness and addressing missing data through full information maximum likelihood, which uses all available data from all participants independently of missing a specific time point [42,43], outperforming other missing data handling methods [42,43]. Both models (intention-to-treat and per-protocol) were adjusted for age, gender and discharge time, as time-invariant covariates. No change was estimated for the subcohort with moderate anxiety (GAD-7 ≥ 10) and productivity impairment since the model did not converge (possibly due to the heterogeneity of recovery trajectories and limited sample size). Additionally, an LGCA subgroup analysis was performed to evaluate potential differences between historically deployed and non-deployed Veterans. All models were estimated with a robust sandwich estimator for standard errors. Estimation of model fit was assessed through chi-squared test, root mean square error of approximation (RMSEA), confirmatory fit index (CFI), and standardized root mean square residual (SRMR) [44,45].

All statistical analyses were conducted using commercially available software (SPSS v22, IBM, Armonk, NY, USA) and R (version 4.2.2, R Foundation for Statistical Computing). The level of significance was set at *p* < 0.05 for all tests.

## 3. Results

**Figure 1:**
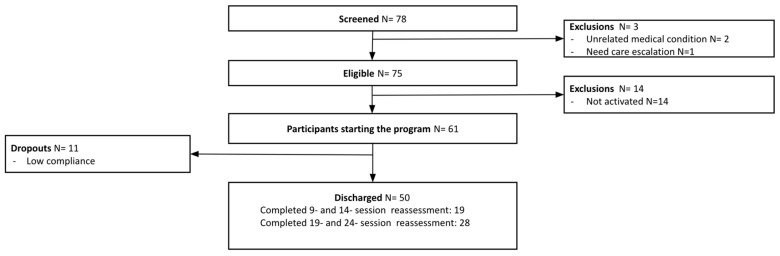
Veteran pilot study flow chart.

For this pilot study, 78 Veterans were screened (Figure 1). Three were excluded due to unsuitable medical conditions. Of the 75 eligible participants, an additional 14 were excluded for failing to activate the program. The program started with 61 Veterans and had a completion rate of 82.0% (50 out of 61 Veterans).

### 3.1. Baseline Characteristics

Veterans’ experience originated from different military branches, and they were predominantly men (55.7%, N = 34), non-Hispanic white (62.3%, N = 38), middle-aged (45–60 years old: 54.1%, N = 33), overweight (83.6% overweight or obese; mean BMI: 30.1, SD 6.0), with higher education (Bachelor’s and Graduate degree: 65.6%, N = 40), and from urban areas (85.2%, N = 52) (Table 1). Most Veterans had been deployed (59%, N = 36). The cohort was mainly employed (50.9%, N = 31) or retired (34.4%, N = 21), with a small proportion of Veterans reporting non-employment (13.1%, N = 8).

Most frequent symptomatic body areas included low back (34.4%, N = 21), knee (18%, N = 11), and hip (16.4%, N = 10). The majority of patients reported symptoms of mental distress (GAD-7 ≥ 5: 63.9%, N = 39; PHQ-9 ≥ 5: 60.7%, N = 37), of which 34.4% (N = 21) reported clinical depression [46] (PHQ-9 ≥ 10) and 26.2% (N=16) reported clinical anxiety [32] (GAD-7 ≥ 10). Employed Veterans (N = 31) reported an average of 24.1 (SD 24.0) overall productivity impairment. The whole cohort reported high impairment in the activities of daily living, with an average score of 43.9 (SD 26.9).

No significant baseline differences were found between completers (N = 50) and non-completers (N = 11).

**Table 1:** Baseline characteristics of study patients for the entire cohort and for completers.

**Characteristic**	**Entire Cohort** **(N = 61)**	**Completers** **(N = 50)**	**Non-Completers** **(N = 11)**	***p*** **-Value**
**Age (years), mean (SD)**	49.7 (10.4)	46.6 (13.8)	50.4 (9.6)	0.247
**Age categories (years), N (%):**				0.119
25–44	19 (31.1)	13 (26.0)	6 (54.5)	
45–60	33 (54.1)	30 (60.0)	3 (27.3)	
>60	9 (14.8)	7 (14.0)	2 (18.2)	
**Gender, N (%)**				0.097
Woman	26 (42.6)	22 (44.0)	4 (36.4)	
Man	34 (55.7)	28 (56.0)	6 (54.5)	
Non-binary	1 (1.6)	0	1 (9.1)	
**BMI (kg/m^2^), mean (SD)**	30.1 (6.0)	30.2 (6.2)	30.0 (5.4)	0.924
**BMI categories (kg/m^2^), N (%):**				0.307
Underweight (<18.5)	2 (3.3)	1 (2.0)	1 (9.1)	
Normal (18.5–25)	8 (13.1)	8 (16.0)	0	
Overweight (≥25–30)	23 (37.7)	19 (38.0)	4 (36.4)	
Obese (≥30–40)	24 (39.3)	18 (36.0)	6 (54.5)	
Morbidly obese (>40)	4 (6.6)	4 (8.0)	0	
**Race/ethnicity, N (%):**				0.264
Asian	2 (3.3)	2 (4.0)	0	
Black	7 (11.5)	6 (12.0)	1 (9.1)	
Hispanic	9 (14.8)	8 (16.0)	1 (9.1)	
Non-Hispanic White	38 (62.3)	30 (60.0)	8 (72.7)	
Other	4 (6.6)	4 (8.0)	0	
Prefer not to specify or NA	1 (1.6)	0	1 (9.1)	
**Education level, N (%):**				0.088
High school diploma	2 (3.3)	1 (2.0)	1 (9.1)	
Some college	19 (31.1)	14 (28.0)	5 (45.5)	
Bachelor’s degree	22 (36.1)	17 (34.0)	5 (42.5)	
Graduate degree	18 (29.5)	18 (36.0)	0	
**Geographic location, N (%):**				0.196
Urban	52 (85.2)	44 (88.0)	8 (72.7)	
Rural	9 (14.8)	6 (12.0)	3 (27.3)	
**Employment status, N (%):**				0.071
Full-time job	27 (44.3)	24 (48.0)	3 (27.3)	
Part-time job	4 (6.6)	2 (4.0)	2 (18.2)	
Retired	21 (34.4)	18 (36.0)	3 (27.3)	
Not employed	8 (13.1)	6 (12.0)	2 (18.2)	
Prefer not to specify or NA	1 (1.6)	0	1 (9.1)	
**Branch of Service, N (%) ^a^:**				0.815
Air Force	10 (17.9)	8 (17.4)	2 (20.0)	
Army	26 (46.4)	22 (47.8)	4 (40.0)	
Coast Guard	2 (3.6)	1 (2.2)	1 (10.0)	
Marine Corps	6 (10.7)	5 (10.9)	1 (10.0)	
Navy	12 (21.4)	10 (21.7)	2 (20.0)	
**Deployment, N (%):**				
Yes	36 (59.0)	30 (60.0)	6 (54.5)	0.739
**Former Military Officer, N (%) ^b^:**				
Yes	10 (18.2)	9 (19.6)	1 (11.1)	0.548
**Clinical data, mean (SD)**				
**Symptomatic anatomical area, N (%):**				0.476
Ankle	2 (3.3)	1 (2.0)	1 (9.1)	
Elbow	0	0	0	
Hip	10 (16.4)	9 (18.0)	1 (9.1)	
Knee	11 (18.0)	9 (18.0)	2 (18.2)	
Low back	21 (34.4)	15 (30.0)	6 (54.5)	
Neck	7 (11.5)	7 (14.0)	0	
Shoulder	8 (13.1)	7 (14.0)	1 (9.1)	
Wrist or hand	2 (3.3)	2 (4.0)	0	
**Acuity ^g^, N (%):**				0.500
Acute	2 (3.3)	2 (4.0)	0	
Chronic	59 (96.7)	48 (96.0)	11 (100)	
**Pain intensity**	5.7 (1.8)	5.7 (1.9)	5.4 (1.6)	0.558
**GAD-7**	6.3 (5.4)	6.5 (5.6)	5.4 (4.9)	0.521
**GAD-7 ≥ 5 ^c^**	9.5 (4.1)	9.6 (4.3)	9.2 (2.7)	0.811
**GAD-7 ≥ 10 ^d^**	13.6 (3.1)	14.1 (3.2)	11.3 (1.2)	0.175
**PHQ-9**	7.3 (6.8)	7.5 (7.0)	6.3 (6.2)	0.594
**PHQ-9 ≥ 5 ^e^**	11.7 (5.1)	12.1 (5.2)	9.9 (4.7)	0.304
**PHQ-9 ≥ 10 ^f^**	15.1 (4.2)	15.2 (4.5)	14.7 (2.1)	0.854
**WPAI overall**	24.1 (24.0)	21.8 (21.4)	33.2 (33.4)	0.306
**WPAI work**	22.0 (22.0)	20.4 (20.1)	28.3 (29.9)	0.441
**WPAI time**	3.6 (9.3)	2.1 (6.2)	9.5 (16.4)	0.081
**WPAI activity**	43.9 (26.9)	45.0 (27.5)	39.1 (24.3)	0.513

Abbreviations: BMI, body mass index; GAD-7, Generalized Anxiety Disorder 7-item scale; NA, not available; PHQ-9, Patient Health 9-item questionnaire; WPAI, Work Productivity and Activity Impairment Questionnaire. Notes: ^a^: Missing N = 5 (completers: N = 1; non-completers: N = 4). ^b^: Missing N = 6 (completers: N = 4; non-completers: N = 2). ^c^: N = 39 (completers: N = 33; non-completers: N = 6). ^d^: N = 16 (completers: N = 13; non-completers: N = 3). ^e^: N = 37 (completers: N = 30; non-completers: N = 7). ^f^: N = 21 (completers: N = 18; non-completers: N = 3). ^g^: chronic pain was defined as persistent or recurrent pain lasting longer than 3 months [47].

### 3.2. Clinical Outcomes

Given the pilot nature of this study, per protocol analyses (Table 2) are primarily reported to better understand the intervention effectiveness in an ideal scenario [48]. For comparison, the intention-to-treat analysis is available in Supplementary Table S2. Model fitness for both analyses is presented in Supplementary Tables S3 and S4.

Pain was significantly reduced by program end (mean change 1.98 points, 95%CI 0.13; 3.84, *p* = 0.036). Improvements in mental health were observed in symptomatic individuals, regardless of severity. Patients with at least mild mental distress (GAD or PHQ ≥ 5) reported reductions of 2.25 (95%CI 0.90; 3.61) in anxiety (*p* = 0.001) and of 1.62 (95%CI 0.54; 2.69) in depression (*p* = 0.003). Patients with clinical baseline depression (PHQ-9 ≥ 10) reported a reduction of 1.89 (95%CI 0.41; 3.36) at program end (*p* = 0.012). No change was estimated for the subcohort with clinical anxiety (GAD-7) or productivity impairment since the model did not converge. Among those reporting impairment in daily activities, a decrease of 13.33 (95%CI 1.31; 25.34) was observed at program end.

### 3.3. Engagement

Veterans performed on average 22.3 (SD 16.8) exercise sessions, with completers performing 25.9 sessions (SD 16.6). On average, Veterans interacted 23.4 (SD 10.2) times with the physical therapist via the in-built chat. Treatment satisfaction was high, with a mean of 9.5 (SD 1.0) out of 10.

### 3.4. Safety and Adverse Events

No adverse events related to the intervention were reported.

### 3.5. Subgroup Analysis: Outcomes per Deployment Status

Baseline demographic and clinical characteristics comparing Veterans with and without combat deployment experience are presented in Supplementary Table S5. Veterans with combat deployment experience presented a higher proportion of men (73.5% (N = 25) versus 36.0% (N = 9) of non-deployed Veterans; *p* = 0.025). All other baseline demographic and clinical features were similar between those with and without deployment experience.

Both groups presented similar engagement (Supplementary Table S5), alongside similar recoveries in all clinical outcomes, with no significant differences on mean changes between groups (all: *p* > 0.05, Table 3; Model fitness in Supplementary Table S6).

## 4. Discussion

### 4.1. Main Findings

This pilot study is the first to evaluate the feasibility and clinical outcomes of a completely remote DCP in the management of MSP among Veterans. High engagement levels were observed across the study, reflected by the high completion rate (82%), high number of sessions completed (25.9, SD 16.6), and frequent interactions with PTs (23.4, SD 10.2). Despite the moderate baseline pain level and the frequent comorbidity with mental distress, significant improvements were observed across all clinical outcomes, alongside a very high program satisfaction (9.5/10, SD 1.0). Pain levels were reduced on average by 1.98 points (95%CI 0.13; 3.84, *p* = 0.036). Among those with at least mild mental health distress, improvements were observed in both anxiety (GAD ≥ 5: 2.25, 95%CI 0.90; 3.61, *p* = 0.001) and depression (PHQ-9 ≥ 5: 1.62, 95%CI 0.54; 2.69, *p* = 0.003). Importantly, participants with at least moderate baseline depression (PHQ-9 ≥ 10) ended the program with mild symptoms (8.50 points, 95%CI: 6.49 to 10.51, *p* = 0.012). Veterans also reported a significant recovery in performing activities of daily living at program end (13.33, 95%CI 1.31; 25.34; *p* = 0.030). The above engagement and clinical improvements were observed regardless of deployment experience, suggesting that undergoing this experience did not impact their ability to engage and recover through a fully remote digital setting. These findings suggest a positive effect of the DCP on Veterans’ overall quality of life, and underscore the potential of expanding Veterans’ access to timely MSP management through a DCP, whose implementation may alleviate the burden associated with these conditions.

### 4.2. Comparison with Literature

Despite the evidence supporting physical therapy as a first-line approach in managing MSP, Veterans often face general and specific access challenges that lead them to forgo care or resort to escalated care options, including opioids, injections, or surgical procedures [19]. The present pilot study is the first to focus on a completely remote MSP DCP (incorporating exercise, education and CBT) to evaluate its potential as a valid alternative for the veteran population to address access challenges. The demographic characteristics of this pilot cohort generally align with those described by the US Census Bureau for Veterans [49] and prior research [50], characterized by a majority of white non-Hispanic and middle-aged individuals. Notably, a more balanced gender distribution (42.6% women and 55.7% men) was observed compared to the highly predominant proportion of men in previous veteran studies, which ranged from 70 to 90% [21,22,39,50,51,52]. Additionally, the cohort had a lower representation of those in rural areas when compared with the national average (15% versus 25%) [17].

A promising high completion rate (82.0%) was attained in this study, alongside a very high satisfaction level of 9.5/10 (SD 1.0), suggesting an overall good acceptance of the DCP. Also supporting this trend were the high number of interactions with the PT and of exercise sessions performed. It can be highly challenging for Veterans to comply with such a high number of sessions in traditional in-person settings, especially when considering the psychological burden frequently triggered during traveling among Veterans [53], prevalent transportation barriers [54], and the significant proportion of Veterans that reside in rural areas nationwide [17].

The remote and asynchronous nature of this DCP allows for a wider accessibility and convenience, while maintaining the clinical rigor of receiving real-time biofeedback and continuous monitoring by the assigned PT [23,24]. The success of physical therapy is highly dependent on adherence levels [55,56], and therefore, having interventions that support good engagement levels is particularly important for populations with severe or comorbid conditions, as is often the case among Veterans [6,57]. Consistent with the previously described [6,7], this pilot cohort presented significant pain levels at baseline, which was frequently comorbid with aggravated mental clinical presentations (GAD-7 ≥ 5: 64%; PHQ-9 ≥ 5: 60% and PHQ-9 ≥ 10: 34.4%). Military service frequently contributes to severe mental distress [8], with previous studies reporting a prevalence of 26.6% on major depression [22]. Clinical outcome recovery is frequently impacted negatively among those with aggravated mental distress [5,58]. Herein, significant and meaningful reductions in pain were observed at program end (change 1.98, 95%CI 0.13; 3.84, *p* = 0.036 [37]), outperforming the average reported for in-person [22,38,39,59] or hybrid [21] settings. For instance, Anamkath et al. [39] assessed within the VHA system an in-person 12-week pain rehabilitation program (movement strategies, education, and CBT) and did not find significant improvements in pain. In another study [21] (N = 221), evaluating a remote interdisciplinary program primarily focused on mental health (video calls conducted by pain physicians, physical therapists, or nurses) for Veterans with chronic MSP, no significant changes in pain were reported following the intervention, not even at a 26-week follow-up.

Importantly, despite the aggravated baseline mental symptomatology, this veteran cohort reported significant improvements in both anxiety and depression (*p* = 0.001 and *p* = 0.003), which was even more evident when considering Veterans with severe symptomatology (GAD ≥ 5: *p* = 0.001, PHQ-9 ≥ 10: *p* = 0.012). Prior studies evaluating interdisciplinary pain management programs including physical therapists have also reported significant improvements in these outcomes after the intervention [21,51,52]; however, the use of different measures precludes direct comparisons with our study.

While the heterogeneity of recovery trajectories of work productivity impairment and the small sample size prevented model convergence, new insights were obtained in the impact of the program in restoring the ability to perform activities of daily living. This cohort reported a much higher baseline impairment of activities of daily living than that observed previously in other non-veteran cohorts [26,27]. Regardless of the aggravated impairment, significant improvements (*p* = 0.030) were observed across the cohort, which is consistent with other studies focused on physical therapy interventions [20,21,39,59].

Deployment has been recognized as a contributing factor for chronicity, higher pain levels, anxiety, and depression [60,61], contrasting the baseline clinical presentations described herein, where no significant differences were found. The subgroups limited sample size and the self-referred approach to enrollment may have contributed to a different pattern. Nonetheless, Veterans with or without deployment reported improvements in outcomes, not significantly different between groups. This finding hints towards the potential of implementing a DCP to manage MSP despite historical deployment experiences, opening new avenues for future studies.

Overall, these promising results might suggest the potential of implementing innovative care delivery solutions to provide easy and timely access to evidence-based care for Veterans with MSP.

### 4.3. Strengths and Limitations

The major strengths of our trial include (1) the novelty of assessing a completely remote MSP multimodal rehabilitation program in a veteran population, which has not been explored before; (2) the real-world context of this study, which may provide a more accurate reflection of the outcomes [62] and a basis for future studies comprising large sample sizes to focus on the transferability of these findings in veteran subpopulations; (3) the use of a diverse array of validated metrics encompassing both physical and psychological domains. Finally, this study offers pioneering insights which may serve as a foundational basis for subsequent larger studies.

The study is not exempt from limitations. The pilot nature of the study, combined with the absence of a control group, results in a limited sample size that restricts the generalizability of the findings and prevents the establishment of causality. Nevertheless, 96.7% of the cohort suffers from chronic MSP, which typically does not tend to recover spontaneously throughout its natural course. Additionally, the lack of long-term follow-up prevents the assessment of the sustainability of the observed improvements. Since participants were self-referred, the cohort may not depict the entire veteran population, even though this pilot cohort encompassed Veterans from several branches, military ranks, deployment experience and age categories. Considering the limited health information that this study could assess, we cannot rule out the possibility of the influence of important confounders not captured in the analysis (e.g., post-traumatic stress disorder, medication intake, other comorbidities). These limitations highlight important future steps for further investigation in larger cohorts, for which the present results are key to support its planning.

## 5. Conclusions

This is the first study demonstrating the feasibility of delivering a completely remote, multimodal DCP for MSP in a veteran population. The high acceptance of the DCP by Veterans was evidenced by their significant engagement and satisfaction with the program. The significant improvements in all clinical outcomes, including pain, mental health, and daily activities (regardless of deployment experience), underscore the potential for expanding the application of DPCs in managing MSP among veterans. Altogether, this study constitutes important groundwork for future studies in the veteran population.

## Figures and Tables

**Table 2:** Model estimates of clinical outcome measures following a per-protocol approach.

Outcome, Mean (95%CI)	N	Baseline	End-Score	Mean Change	*p*-Value
Pain Level	50	5.71 (5.21; 6.21)	3.73 (1.90; 5.55)	1.98 (0.13; 3.84)	*0.036*
GAD-7 > 0	36	9.15 (7.80; 10.50)	7.05 (5.71; 8.39)	2.10 (0.86; 3.34)	*0.001*
GAD-7 ≥ 5	33	9.77 (8.44; 11.11)	7.35 (6.17; 8.88)	2.25 (0.90; 3.61)	*0.001*
PHQ-9 > 0	34	8.17 (6.54; 9.80)	6.71 (5.16; 8.26)	1.46 (0.55; 2.37)	*0.002*
PHQ-9 ≥ 5	30	9.15 (7.61; 10.68)	7.53 (5.95; 9.11)	1.62 (0.54; 2.69)	0.003
PHQ-9 ≥ 10	18	10.38 (8.70; 12.07)	8.50 (6.49; 10.51)	1.89 (0.41; 3.36)	*0.012*
WPAI activities > 0	46	46.53 (39.67; 53.40)	33.21 (22.51; 43.90)	13.33 (1.31; 25.34)	*0.030*

Abbreviations: GAD-7, Generalized Anxiety Disorder 7-item scale; PHQ-9, Patient Health 9-item questionnaire; WPAI, Work Productivity and Activity Impairment Questionnaire. Notes: Significant *p*-values are italicized. Models for GAD-7 ≥ 10 and productivity impairment did not converge.

**Table 3:** Subgroup analysis: clinical outcomes estimates stratified by deployment.

Outcome	Deployment	N	Baseline	End-Score	Mean Change	Mean Difference between Groups	*p*-Value between Groups
Pain	No	20	5.66 (4.86; 6.65)	3.83 (2.38; 5.29)	1.83 (0.20; 3.46)	0.28 (−1.38; 1.93)	0.744
	Yes	30	5.75 (5.12; 6.38)	4.20 (2.16; 6.23)	1.55 (0; 3.64)		
GAD-7 > 0	No	15	9.38 (7.20; 11.56)	6.44 (4.49; 8.39)	2.94 (1.23; 4.65)	1.38 (−0.58; 3.33)	0.167
	Yes	21	8.98 (7.25; 10.71)	7.42 (5.62; 9.23)	1.56 (0.11; 3.01)		
GAD-7 ≥ 5	No	19	9.97 (7.87; 12.06)	7.22 (5.84; 8.59)	2.75 (0.95; 4.55)	0.86 (−1.20; 2.93)	0.413
	Yes	14	9.63 (7.95; 11.31)	7.74 (5.84; 9.64)	1.89 (0.28; 3.50)		
PHQ-9 > 0	No	14	8.93 (6.51; 11.35)	6.79 (4.52; 9.05)	2.15 (0.73; 3.57)	1.10 (−0.76; 2.97)	0.245
	Yes	20	7.70 (5.39; 10.00)	6.65 (4.47; 8.84)	1.04 (0.09; 2.18)		
PHQ-9 ≥ 5	No	14	8.94 (6.55; 11.32)	6.77 (4.62; 8.92)	2.17 (0.71; 3.62)	0.89 (−1.16; 2.93)	0.396
	Yes	16	9.45 (7.33; 11.57)	8.17 (6.01; 10.33)	1.28 (0.13; 2.69)		
PHQ-9 ≥ 10	No	9	10.33 (7.32; 13.35)	7.62 (4.39; 10.85)	2.71 (0.72; 4.70)	1.26 (−1.44; 3.96)	0.361
	Yes	9	10.52 (7.89; 13.15)	9.06 (6.11; 12.02)	1.45 (0.29; 3.19)		
WPAI activities > 0	No	19	47.55 (37.03; 58.06)	36.79 (21.55; 52.04)	10.75 (6.52; 28.03)	−2.37 (−24.80; 20.06)	0.836
	Yes	27	45.30 (37.01; 53.60)	32.18 (17.94; 46.42)	13.12 (2.64; 28.88)		

Abbreviations: GAD-7, Generalized Anxiety Disorder 7-item scale; PHQ-9, Patient Health 9-item questionnaire; WPAI, Work Productivity and Activity Impairment Questionnaire.

## Data Availability

The data presented in this study are available on request from the corresponding author. The data are not publicly available due to privacy restrictions.

## Supplementary Materials

The following supporting information can be downloaded at: https://www.mdpi.com/article/10.3390/healthcare12151518/s1, Table S1. Description of the exercise prescription; Table S2. Model estimates of clinical outcome measures: intention-to-treat; Table S3. Model fitness of the LGCA: per protocol; Table S4. Model fitness of the LGCA: intention-to-treat; Table S5. Baseline characteristics and engagement metrics of study patients stratified by deployment; Table S6. Model fitness of the subgroup LGCA stratified by deployment status: per protocol.
