# Supplementary material for: Transforming Veteran Rehabilitation Care: Learnings from a Remote Digital Approach for Musculoskeletal Pain
Source: Healthcare (Basel). 2024 Jul 31;12(15):1518. doi: 10.3390/healthcare12151518 (PMC11311802; doi:10.3390/healthcare12151518)
Supplement: Supplementary file 1 [file healthcare-12-01518-s001.zip › healthcare-3113434-supplementary.pdf]

Supplementary Materials

Transforming Veteran rehabilitation care: learnings from a remote digital approach for musculoskeletal pain

Anabela C. Areias, Dan Doverspike, Dan Brostek, Dora Janela, Mike Erwin, John Pinter, James Ficke, Fabíola Costa

Supplementary Table S1. Description of the exercise prescription.

Phases and Main goals	Intervention description
**Initial Phase**<br><br>Goals:<br>• Decrease symptoms and inflammatory signs<br>• Gradual exposure to avoided movements<br>• Improve range of motion (passive and active)<br>• Improve muscular strength and endurance<br>• Improve motor control and stability<br>• Provide knowledge on the condition, contributing factors and the role of exercise	Aim for at least 1-2 sets of:<br>• Articular mobility exercises<br>• Strengthening exercises<br>• Active and passive stretching exercises
**Intermediate Phase**<br><br>Goals:<br>• Improve range of motion<br>• Increase global muscular and aerobic capacity<br>• Increase time of exposure to painful movements<br>• Gradual exposure to daily activities that elicit symptoms	Increase repetitions and sets or add external load (bands and/or free weights) of the prescribed exercises;<br><br>Add more demanding exercises<br><br>Progressively increase average session time<br><br>Increase exercise complexity and aim for at least 2-3 sets of:<br>• Articular mobility exercises (e.g. standing trunk movements)<br>• Strengthening exercises (e.g. isometric wall squat; plank)<br>• Active and passive stretching exercises (standing anterior thigh stretch)

**Late Phase**<br><br>Goals:<br>● Functional restoration of range of motion<br>● Return to daily activities<br>● Increase time of exposure to daily activities that elicit symptoms<br>● Foster independence	Add or increase external load of prescribed exercises (bands and/or free weights)<br><br>Increase complexity and add single leg stance exercises:<br>● Articular mobility exercises (e.g. single leg knee to chest)<br>● Strengthening exercises (e.g. plank with trunk movement or with hand movement; lunges)<br>● Active and controlled stretching exercises

Notes:
In addition to anamnesis, physical assessment comprised the evaluation of movement patterns, addressing compensations/difficulties during movements execution, and active and passive range of motion in uni-joint and multi-joint movements (measured by the motion tracking technology).
The exercise prescription was tailored according to the initial assessment and individual patient progress during the study.
The following parameters of the exercise prescription were adjusted by the physical therapist according to the patient's evolution: range of motion, number of exercises, number of sets and repetitions and the type of exercise. Alongside assessment and communication between patients and PT, the performance (namely the range of motion, execution, movement compensations and skipped exercises) and the level of pain and fatigue during exercises reported by the patient were taken in consideration for the intervention decision making.

Supplementary Table S2. Model estimates of clinical outcome measures: intention-to-treat.

Outcome, mean (95%CI)	N	Baseline	End-score	Mean Change	% Change	P value
**Pain Level**	61	5.53 (5.08; 5.97)	3.54 (2.92; 4.16)	-1.99 (-2.66; -1.32)	35.9%	*<0.001*
**GAD-7 >0**	43	9.02 (7.87; 10.17)	6.90 (5.44; 8.35)	-2.12 (-3.44; -0.81)	23.5%	*0.002*
**GAD-7 ≥5**	39	9.52 (8.40; 10.63)	7.30 (5.78; 8.82)	-2.22 (-3.62; -0.82)	23.3%	*0.002*
**PHQ-9 >0**	41	7.97 (6.54; 9.41)	6.61 (5.12; 8.10)	-1.36 (-2.25; -0.47)	17.1%	*0.003*
**PHQ-9 ≥5**	37	8.74 (7.38; 10.11)	7.32 (5.74; 8.90)	-1.43 (-2.50; -0.36)	16.4%	0.009
**PHQ-9 ≥10**	21	10.21 (8.71; 11.72)	8.34 (6.37; 10.31)	-1.87 (-3.36; -0.39)	18.3%	*0.013*
**WPAI activities >0**	56	45.84 (39.83; 51.85)	31.11 (19.53; 42.69)	-14.73 (-27.59; -1.87)	32.1%	*0.025*

Abbreviations: GAD-7, Generalized Anxiety Disorder 7-item scale; PHQ-9, Patient Health 9-item questionnaire; WPAI, Work Productivity and Activity Impairment Questionnaire.

Supplementary Table S3. Model fitness of the LGCA: per protocol.

Outcome, mean (95%CI)	CFI	SRMR	RMSEA
**Pain Level**	0.99	0.212	0.034
**GAD-7 >0**	1.00	0.075	0.000
**GAD-7 ≥5**	1.00	0.124	0.000
**PHQ-9 >0**	1.00	0.081	0.000
**PHQ-9 ≥5**	1.000	0.108	0.000
**PHQ-9 ≥10**	1.000	0.100	0.000
**WPAI activities >0**	1.000	0.058	0.000

Notes: Model appropriateness was assessed using standardized root mean square residual (SRMR), root mean square error approximation (RMSEA), and confirmatory fit index (CFI), applying the following cut-off criteria: CFI = close to 0.95; RMSEA = close to 0.06 and SRMR = close to 0.08.

Supplementary Table S4. Model fitness of the LGCA: intention-to-treat.

Outcome, mean (95%CI)	CFI	SRMR	RMSEA
**Pain Level**	0.96	0.113	0.061
**GAD-7 >0**	1.00	0.077	0.000
**GAD-7 ≥5**	1.00	0.137	0.000
**PHQ-9 >0**	1.00	0.086	0.000
**PHQ-9 ≥5**	1.00	0.114	0.000
**PHQ-9 ≥10**	1.00	0.101	0.000
**WPAI activities >0**	1.00	0.055	0.000

Notes: Model appropriateness was assessed using standardized root mean square residual (SRMR), root mean square error approximation (RMSEA), and confirmatory fit index (CFI), applying the following cut-off criteria: CFI = close to 0.95; RMSEA = close to 0.06 and SRMR = close to 0.08.

Supplementary Table S5. Baseline characteristics and engagement metrics of study patients stratified by deployment.

Characteristic	Deployed (N=36)	Non-deployed (N=25)	*P* value
**Age (years), mean (SD)**			
**Age categories (years), N (%):**	11 (30.6)	8 (32.0)	.579
25-44	21 (63.6)	12 (48.0)	
45-60	4 (11.1)	5 (20.0)	
> 60			
**Gender, N (%)**			*.025*
Woman	11 (30.6)	15 (60.0)	
Man	25 (73.5)	9 (36.0)	
Non-binary	0 (0.0)	1 (4.0)	
**BMI (kg/m²), mean (SD)**			
**BMI categories (kg/m²), N (%):**			.218
Underweight (<18.5)	1 (2.8)	1 (4.0)	
Normal (18.5-25)	7 (19.4)	1 (4.0)	
Overweight (≥25-30)	10 (27.8)	13 (52.0)	
Obese (≥30-40)	16 (44.4)	8 (32.0)	
Morbidly obese (>40)	2 (5.6)	2 (8.0)	
**Race/ethnicity, N (%):**			.824
Asian	1 (2.8)	1 (4.0)	
Black	3 (8.3)	4 (16.0)	
Hispanic	6 (16.7)	3 (12.0)	
Non-Hispanic White	22 (61.1)	16 (64.0)	
Other	3 (8.3)	0 (0.0)	
Prefer not to specify or NA	1 (2.8)	1 (4.0)	
**Education level, N (%):**			0.384
High school diploma	0 (0)	2 (8.0)	
Some college	12 (33.3)	7 (28.0)	
Bachelor's degree	13 (36.1)	9 (36.0)	
Graduate degree	11 (30.6)	7 (28.0)	
**Geographic location, N (%)[a]:**			0.069
Urban	28 (77.8)	24 (96.0)	
Rural	8 (22.2)	1 (4.0)	
**Employment status, N (%):**			0.170
Full-time job	18 (50.0)	9 (36.0)	
Part-time job	3 (8.3)	1 (4.0)	
Retired	13 (36.1)	6 (24.0)	
Not employed	2 (5.6)	1 (4.0)	
Prefer not to specify or NA	0 (0)	8 (32.0)	
**Branch of Service, N (%)[b]:**			.145
Air Force	6 (17.1)	4 (19.0)	
Army	18 (51.4)	8 (38.1)	
Coast Guard	0 (0)	2 (9.5)	
Marine Corps	2 (5.7)	4 (19.0)	

Navy	9 (25.7)	3 (14.3)	
**Former Military Officer, N (%)[c]:**			
Yes	7 (21.2)	3 (13.6)	.475
**Clinical data, mean (SD)**			
**Symptomatic anatomical area, N (%):**			0.602
Ankle	1 (2.8)	1 (4.0)	
Hip	4 (11.1)	6 (24.0)	
Knee	8 (22.2)	3 (12.0)	
Low back	12 (33.3)	9 (36.0)	
Neck	5 (13.9)	2 (8.0)	
Shoulder	4 (11.1)	4 (16.0)	
Wrist or hand	2 (5.6)	0 (0)	
**Acuity, N (%):**			.231
Acute	2 (5.6)	0 (0.0)	
Chronic	34 (94.4)	25 (42.4)	
**Pain intensity**	5.75 (1.8)	5.52 (1.9)	*.629*
**GAD-7**	6.14 (5.5)	6.42 (5.5)	.748
**GAD-7 ≥5[d]**	9.55 (4.18)	9.53 (4.0)	.990
**GAD-7 ≥10[e]**	13.4 (3.0)	13.81 (3.5)	.797
**PHQ-9**	6.42 (6.4)	8.52 (7.4)	.240
**PHQ-9 ≥5[f]**	10.95 (4.9)	12.53 (5.4)	.358
**PHQ-9 ≥10[g]**	14.36 (3.9)	15.9 (4.5)	.416
**WPAI overall**	28.5 (27.3)	16.5 (15.3)	.191
**WPAI work**	26.32 (25.4)	14.55 (12.1)	.162
**WPAI time**	4.06 (10.3)	2.67 (7.5)	.701
**WPAI activity**	42.8 (27.2)	45.6 (26.5)	.690
**Engagement metrics, mean (SD)**			
**Total sessions completed**	23.6 (13.1)	29.5 (20.6)	.222
**Total interactions**	22.3 (8.3)	25.1 (12.6)	.362
**Program satisfaction[h]**	9.3 (1.1)	9.9 (0.4)	.056

Abbreviations: BMI, body mass index; GAD-7, Generalized Anxiety Disorder 7-item scale; NA, not available; PHQ-9, Patient Health 9-item questionnaire; WPAI, Work Productivity and Activity Impairment Questionnaire

Notes: Significant P-values are italicized.

a: Fisher's exact test

b: N=56 (deployed: N=35; non-deployed: N=21)

c: N=55 (deployed: N=33; non-deployed: N=22)

d: N=39 (deployed: N=21; non-deployed: N=18)

e: N=16 (deployed: N=10; non-deployed: N=6)

f: N=37 (deployed: N=20; non-deployed: N=17)

g: N=21 (deployed: N=11; non-deployed: N=10)

h: N=24 (deployed: N=16; non-deployed: N=8)

Supplementary Table S6. Model fitness of the subgroup LGCA stratified by deployment status: per protocol.

Outcome, mean (95%CI)	CFI	SRMR	RMSEA
**Pain Level**	1.000	0.218	0.000
**GAD-7 >0**	1.000	0.083	0.000
**GAD-7 ≥5**	1.00	0.101	0.000
**PHQ-9 >0**	1.00	0.079	0.000
**PHQ-9 ≥5**	0.996	0.108	0.043
**PHQ-9 ≥10**	1.000	0.103	0.000
**WPAI activities >0**	0.808	0.128	0.058

Notes: Model appropriateness was assessed using standardized root mean square residual (SRMR), root mean square error approximation (RMSEA), and confirmatory fit index (CFI), applying the following cut-off criteria: CFI = close to 0.95; RMSEA = close to 0.06 and SRMR = close to 0.08.
